# Investigation of an innovative savonius turbine in practice

**DOI:** 10.1038/s41598-025-88544-w

**Published:** 2025-02-26

**Authors:** R. Afify, E. Saber, H. Awad

**Affiliations:** Mechanical Engineering Department, Faculty of Engineering, Arab Academy for Science, Technology and Maritime Transport (AASTMT), Abu Kir, Alexandria, P. O. Box 1029, Miami, Egypt

**Keywords:** Savonius turbine, Vertical axis wind turbine (VAWT), Torque, Power, Applied physics, Fluid dynamics, Engineering

## Abstract

The current study recommends a novel design that includes lightweight flapping gates that open when the convex side is facing the wind source in order to reduce negative torque and increase the efficiency of Savonius wind turbines. Very light leaf or torsional springs can be used to ensure that the gates close smoothly by returning them to their closed state. The efficiency of the turbine is increased when the flapping gates open because there is less drag force and resistive torque applied to the turbine shaft. Two turbines, one with the conventional shape and the other with flapping gates, were created in order to achieve this. The two turbines were then compared to one another under similar circumstances. To collect the necessary readings, an experimental setup with a variety of sensors was developed. Experiments were carried out at various air velocities to calculate the static torque coefficient and power coefficient. It was proved that the new design, which has a higher static torque and power coefficient, is more effective than the conventional design. The power coefficient increases by an average of 25% at middle speed ranges, but only by about 16.914% at higher tip speed ratios.

## Introduction

A wind turbine with a vertical rotor axis is known as a vertical axis wind turbine (VAWT). These turbines are beneficial for use on locations where the wind direction is highly variable since the rotor axis is vertical, meaning that they do not need to be directed into the wind in order to operate. They are especially helpful in urban and residential settings since they are much quieter than horizontal axis wind turbines. However, the added drag that the VAWTs create when their blades rotate into the wind makes them less efficient than the HAWTs. In order to increase the VAWT’s efficiency, attempts are being made to lower its drag coefficient. In recent years, the VAWTs have been tested on a number of factors, including solidity, wind velocity, tip speed ratio, and rotor blade finish. There are two main groups for vertical axis wind turbines: Savonius and Darrieus types of VAWT. By developing a novel geometry for the Savonius blade shape, the current effort aims to increase the performance of Savonius wind turbines.

Savonius wind turbine is an aerodynamic drag type configuration that is relatively suitable for low wind speed. Savonius rotor works due to the difference of drag force acting on the concave and convex parts of its blades. Based on the difference between the drag forces on the blades, Savonius wind turbine has poor aerodynamic performances, Menet [1] and Bhutta et al. [2]. Savonius wind turbine has a number of advantages over other wind turbines such as simplicity in design, ease of fabrication and installation in confined spaces, its operation is independent of wind direction, ability to operate under complex turbulent flows [3], low rotation speed and noise emission [4], and good starting performance [5]. On the other hand, disadvantages of Savonius rotor are: lower efficiency, low rotational speed, fluctuations in the torque during operation of the rotor and difficulty in designing high-power wind turbines. The conventional Savonius turbines have a low power coefficient, around 0.21, compared to other vertical axis turbines. Comprehensive studies were carried out to investigate the effect of various design parameters such as overlap [6], number of blades and number of stages [7–9], and endplates [10] on turbine performance using experimental and numerical methods. Additional types of equipment such as obstacle shielding at the returning blade side [11], curtain at upstream of the rotor turbine [12–15], deflector plate [16], and guide-box tunnel [17] can be used to improve the performance of the Savonius turbine. However, the use of additional equipment will make Savonius rotor more complex. Some researchers investigated the use of a helical-shape blade Savonius rotor [18–24]. It has many advantages such as simple construction, independence of wind direction and a good starting torque at lower wind speeds [19, 20]. Zhao et al. [21] and Damak et al. [22] studied four types of helical rotors with different twisted angles $90^{o}$, $180^{o}$, $270^{o}$ and $360^{o}$. They found that the best performance was obtained by a helical rotor of $180^{o}$ twisted angle. The rotor has some downwind surface parts that are exposed to the wind speed at any rotational angles producing positive torque giving better performance than helical rotors with other twist angles. Darrieus-Savonius rotor was introduced by some researchers [25–32] to have the advantage of high starting torque of Savonius turbine and advantage of Darrieus rotor that is high power coefficient. Combined Savonius-Darrieus type vertical axis wind rotor has better efficiency than the Savonius rotor. There are many combined configurations of turbines and the overlap between Savonius blades increases the performance of the combined turbine. Sahim [33] showed that the gap distance between Savonius and Darrieus blades is an important parameter that affects the performance of the turbine. The flow field around the Savonius rotor is very complex and there is no integrated theoretical system to make a complete analysis and prediction of it. So, many researchers [34–40] carried out numerical simulations verified the results by experiments. Thotla [41] installed a semi-automatic valve device in the blades of Savonius turbine. A hole was made in the blade with a raxine-type cover to cover it on the concave side of the blade, acting as a valve. Under this condition, the rotor operates normally like a conventional Savonius turbine. When the convex side of blade is on the windward side, the raxine-type cover is blown open and the airflow goes through the hole in the convex side to reduce the negative torque of the rotor, resulting in a higher torque.

Numerous techniques have been employed to create novel rotor profiles, according to earlier studies. Table 1 describes how the Savonius turbine’s performance can be improved by altering the traditional profile, either by utilizing auxiliary blades or by altering the circular profile’s angle. The new Savonius profiles derived from parametric investigations are displayed in Table 2. Additionally, as indicated in Table 3, modified Savonius rotors were created using optimization algorithms.

**Table 1:** Modifications on semi-circular Savonius profile.

Authors	Achievement
Mao and Tian [48]	The study indicated that increasing the outer blade arc angle to 160o enhanced the Cp by 8.37%
Abdelaziz et al. [49]	They investigated with changing the outer and inner blade arc angles by adjusting the semicircular profile’s outer (P1) and inner (P2) endpoint positions. Changing the outer and inner blade arc angles to 160o and 20o increased the highest possible Cp by 4.5% and 12.9%, respectively
Driss et al. [50]	Examined how the aerodynamic performance of non-traditional Savonius profiles was impacted by the blade arc angle
Abdelaziz et al. [51]	Studied the impact of adding straight and curved auxiliary blades to the traditional buckets’ inner endpoints in order to enhance aerodynamic performance at the overlap area. Auxiliary blades that were straight or curved increased the maximum Cp by 9% and 8.4%, respectively
Altan et al. [52]	The best Cp was raised by 20% by connecting straight auxiliary blades to the semi-circular Savonius rotor’s outer endpoints
Sharma et al. [53]	Investigated adding two-quarter inner blades located in the outer region of the concave surface of the original buckets, which enhanced the maximum Cp by 8.89%
Sharma et al. [54]	The maximum Cp was increased by 11.34% when two layers of tiny inner blades were used in the present investigation
Al-Ghriybah et al. [55]	Examined how changing the angle of one inner blade layer affected the traditional Savonius rotor’s performance. There was a 17.51% increase in the maximum Cp for a certain angle
Al-Ghriybah et al. [56]	Looked into changing the distance between the blades and adding two layers of inner blades. The best Cp increased by 17.1% when the inner blades were positioned in a particular manner
Haddad et al. [57]	The Bach-type Savonius rotor was studied for the addition of inner blades. The best Cp was increased by 22.39% compared to the original Bach turbine
Patel V, Patel R. [58]	Adding two splitters on the concave corner of the original conventional rotor increased the maximum Cp by 7.3%

**Table 2:** Non-conventional Savonius profiles from parametric studies.

Authors	Achievement
Shashikumar et al. [59]	The semi-circular profile of a hydrokinetic Savonius rotor was divided into two fixed endpoints and a variable point, resulting in an inverted V profile with the variable point’s height changed. At a specific height, the performance improved by 19.3%
Shashikumar et al. [60]	The effect of tapering a hydrokinetic conventional rotor profile was investigated by lowering the lower rotor diameter while keeping the top rotor diameter constant. Using a tapered Savonius profile lowered performance by 5%
Absi et al. [61]	Varying the concave surface of the elliptical shape with a corrugated inner surface improved the maximum Cp by 18%
Tian et al. [62]	A particular equation that specifies the curvature design linking the points was used to fix the positions of three points (two endpoints and the point of maximum height) and investigate changing the bucket profile of the Savonius rotor. In comparison to the semi-circular shape, the maximum power coefficient increased by 10.98% for a specific profile curvature
Mahrous [63]	By altering the blade shape factor, the greatest Cp was increased by 48% in comparison to the traditional Savonius rotor
Roy et al. [64]	Investigated and compared the performance of a newly developed profile with those of classic Savonius profiles

**Table 3:** Novel Savonius profiles from optimization algorithms.

Authors	Achievement
Mohamed et al. [65]	A modified Savonius rotor with three changeable points and two fixed endpoints was created using an optimization library (OPAL). When comparing the adjusted profile to the standard profile, the best Cp increased by 40%
Chan et al. [66]	The traditional profile, which was divided similarly to Ref. [65], was optimized with no overlap ratio (e = 0). The maximum Cp was increased by 33% for the improved rotor
Kerikous et al. [67]	Used OPAL software to adjust the hydraulic Savonius rotor’s blade thickness, increasing the maximum Cp by 12%. Two layers make up each bucket, and each layer was separated into two fixed points and three variable points
Tian et al. [68]	Optimized the Savonius rotor’s profile using the Particle Swarm Optimization (PSO) technique. The optimized rotor’s concave surface had an elliptical profile while its convex surface had a semi-circular profile. The conventional design’s maximum Cp was increased by 4.41% by the changed profile design

The aim of the present study is to introduce a modified novel shape of the Savonius rotor blade to reduce/eliminate the negative torque when the convex side of blade is on the windward side. At a certain value of the wind speed, the negative torque on the convex blade may be converted to a positive torque and the performance of the Savonius turbine can be improved significantly.

### Novelty

The present invention introduces a novel and improved geometry of a Savonius turbine blade, which contains flapping gates that rotate around an axis parallel to the turbine’s axes of rotation. The suggested flap gates are evenly distributed across the blade’s surface area and are as small as possible. At different angles of rotation of the convex blade, the value of the pressure acting on the surface changes, causing the flaps to open and allow air to pass through the holes, but with varying openings depending on the pressure value, the mass of the flap, and the radial distance between the flap and the axis of rotation. Furthermore, the area of air leave from the flap gate is less than the area of air input into it, resulting in a large increase in air speed at the gate’s exit region resulting in the creation of a thrust in the direction of the turbine’s rotation. The thrust helps to raise the positive torque, which improves turbine performance. In some cases, the angle of the convex blade can cause the convex total torque acting on the turbine axis to be positive rather than negative, resulting in a significant improvement in turbine performance and efficiency. The present invention provides a new and improved Savonius wind turbine blade that is easily manufactured and does not require external mechanical assistance to reduce the negative torque produced by the convex blade.

## Materials and methods

### Detailed description of the modified turbine and the method of exploitation

**Fig. 1:**
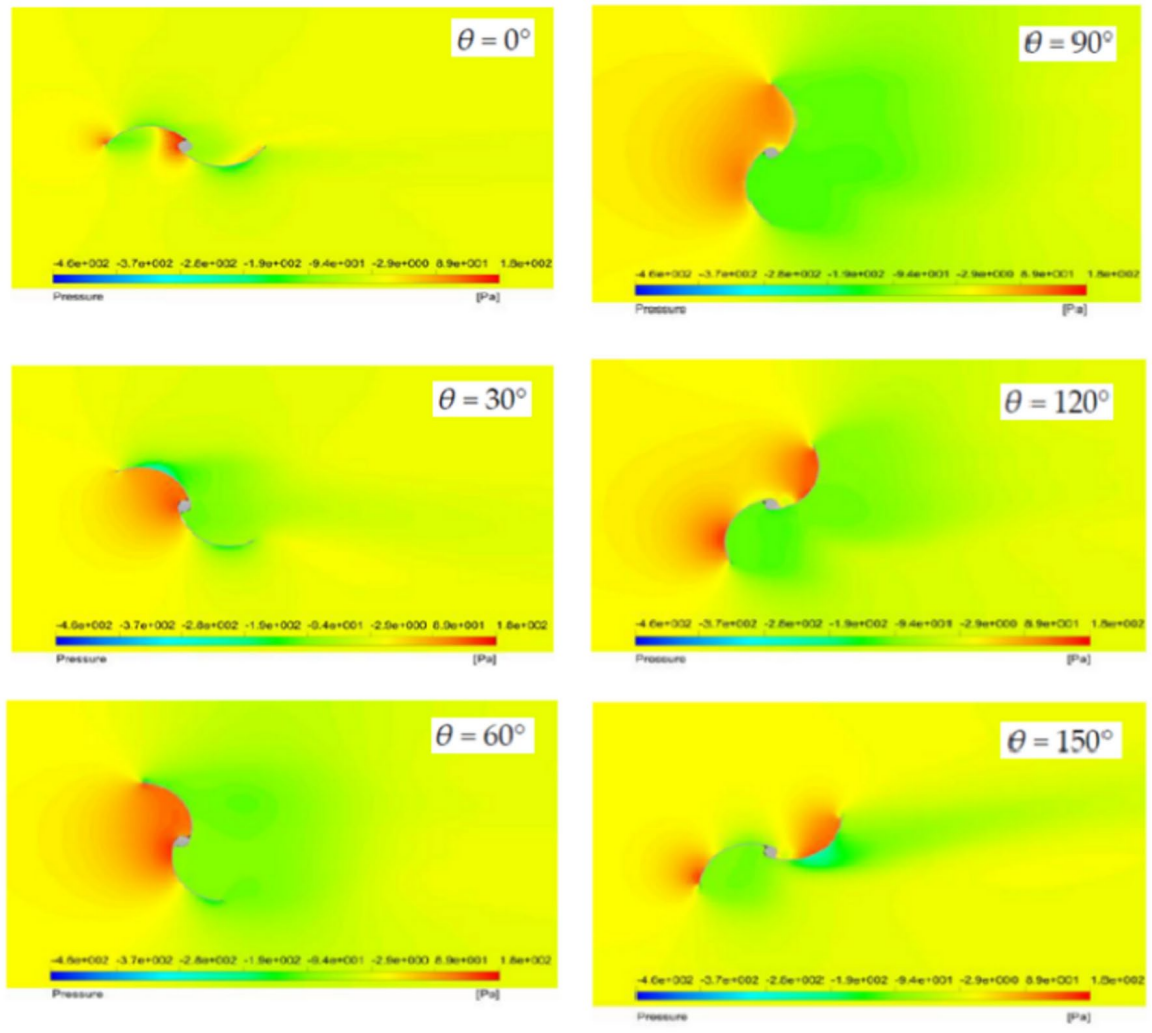
The pressure distribution on the surface of the concave and convex blades of the S-shape Savonius turbine at various rotor position angles ranging from 0 to 150 deg. [42].

The current study presents a novel and better Savonius wind turbine blade that is easy to fabricate and does not require extra mechanical help to lower the convex blade’s negative torque. The latest model also contains a new and improved wind turbine that can be disassembled quickly and easily, takes up minimal space, weighs as little as possible, and is readily transportable. Furthermore, the presence of opening holes in the convex Savonius turbine blade allows air to pass through, which reduces both the drag coefficient and also the drag force acting on it. As a result, the negative torque produced by the convex blade is decreased, enhancing turbine efficiency. According to prior research^42–46^, the pressure distribution on the surface of both the concave and convex blades during the rotation of the Savonius turbine varies with the angles of rotation, as illustrated in Fig. 1. In addition, the presence of flapped-gates in the convex Savonius turbine blade allows air to pass through, which reduces both the drag coefficient and the drag force acting on it. As a result, the negative torque produced by the convex blade is decreased, enhancing turbine efficiency. According to Fig. 1, the maximum pressure occurs near the axis of rotation at angle zero. As the blade angle increases, the area of the surface subjected to maximum pressure increases, until it reaches an angle of around 90 degrees. For angles between 90 and 180 degrees, the area along the axis of rotation gets low pressure values, while the area at the blade’s free edge receives larger pressure values.

**Fig. 2:**
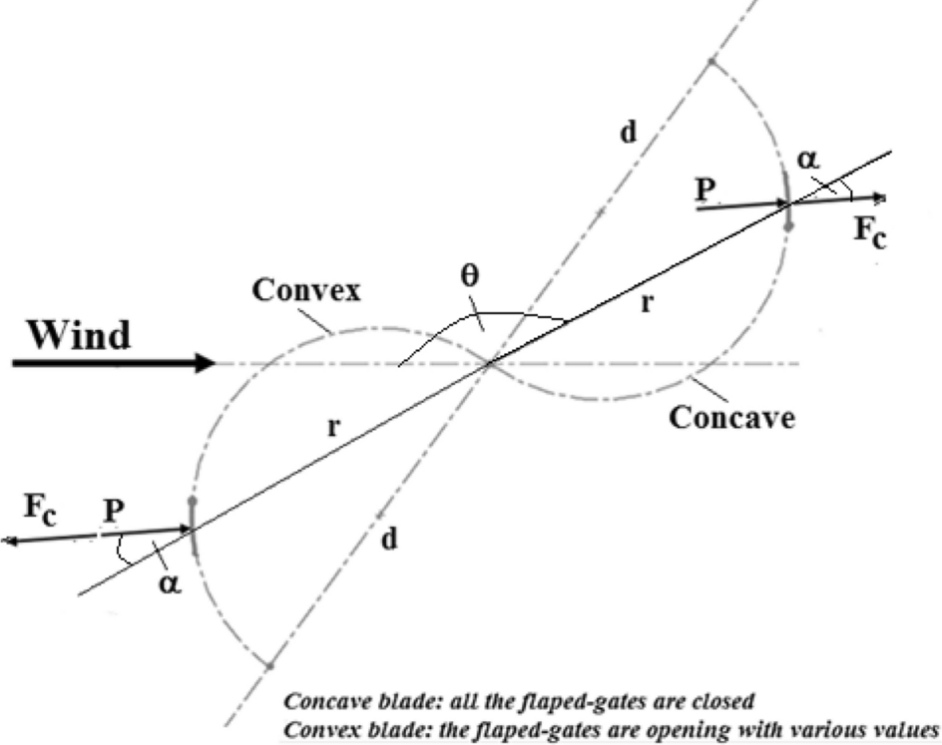
The pressure force and centrifugal force components acting on the flapped-gates in the present turbine rotor’s concave and convex blades at a general rotor position.

As illustrated in Fig. 2, each flap of the present turbine is subjected to the pressure force P and the centrifugal force component $F_{c}$ as the turbine rotates. Because the concave blade’s pressure and centrifugal force component are in the same direction, the flapping gate remains closed. The gate flap in the convex blade is subjected to both pressure and centrifugal forces, but in different directions. The pressure force opens the flapping gate, while the centrifugal force closes it.

For the greatest advantage, the suggested flap gates are distributed throughout the blade’s surface area and are as small as possible. At different angles of rotation of the convex blade, the value of the pressure acting on the surface changes, causing the flaps to open and allowing air to pass through the holes, but with varying openings depending on the pressure value, the mass of the flap, and the radial distance between the flap and the axis of rotation. Figure 2 shows the pressure force P and centrifugal force component acting on a flap in both concave and convex blades. The pressure force and centrifugal force components are $P = p\,A_{f}$ and $F_{c} = m\,r\,\omega^{2} \,\cos \,(\alpha ) = m\,\omega^{2} \,d\,\cos^{2} \,(\alpha )$. p is the average pressure value working on the flap surface area, while P is the pressure force acting on the flap surface area $A_{f}$. m is the flap mass, $\omega$ is the turbine’s angular speed in rad/s, d is the half-cylinder blade diameter, and $\alpha$ is the flap position angle. For the convex blade, when the pressure force P exceeds the centrifugal force component $F_{c}$, the net force acting on the flap surface opens the flapped gate, whereas when the centrifugal force component $F_{c}$ overcomes the pressure force P, the flapped gate closes. The characteristics of any flapped gate that opens at a specific convex blade position are determined by the pressure distribution values at the flap’s location, the flap’s surface area, mass, and position angle, which define the position of the flapped gate for a specific blade diameter (d) at a specific turbine rotation speed. Every flapped-gate opening on the convex blade surface has its own significance and is unlike any other flapped gate. By opening the gates, the drag force on the blade decreases, which lowers the quantity of negative torque produced at the turbine’s rotational axis. As a result, minimizing the negative torque improves both the turbine’s efficiency and the net torque acting on its axis.

**Fig. 3:**
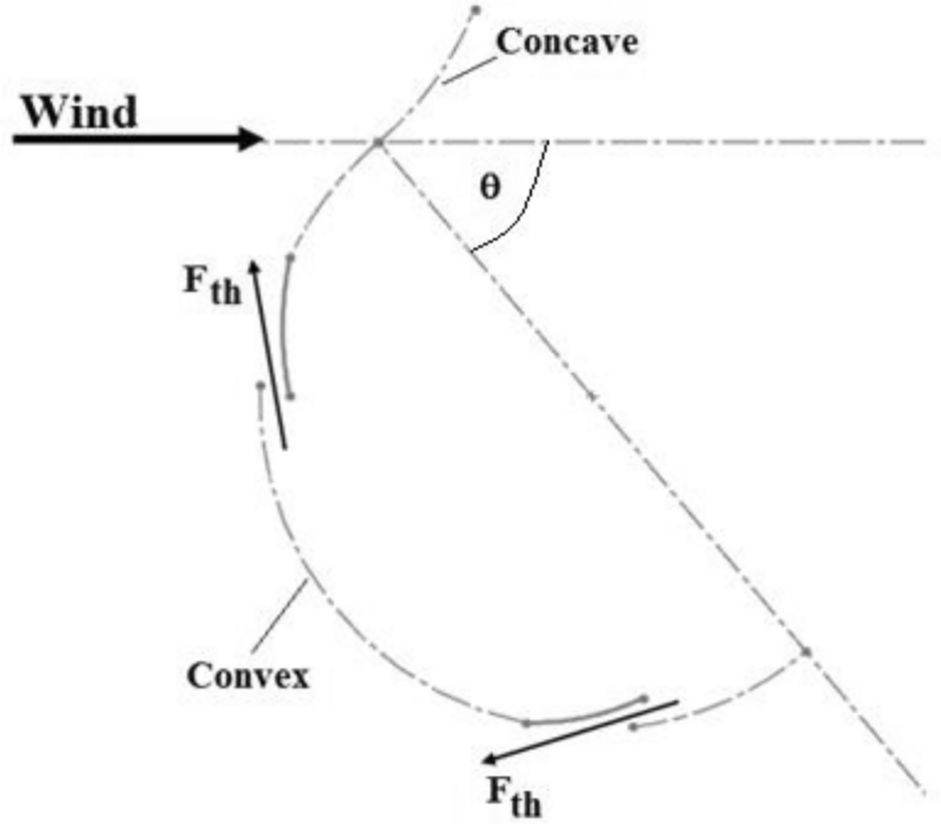
Thrust forces generated based on the position of the flapped-gate on the convex blade surface.

**Fig. 4:**
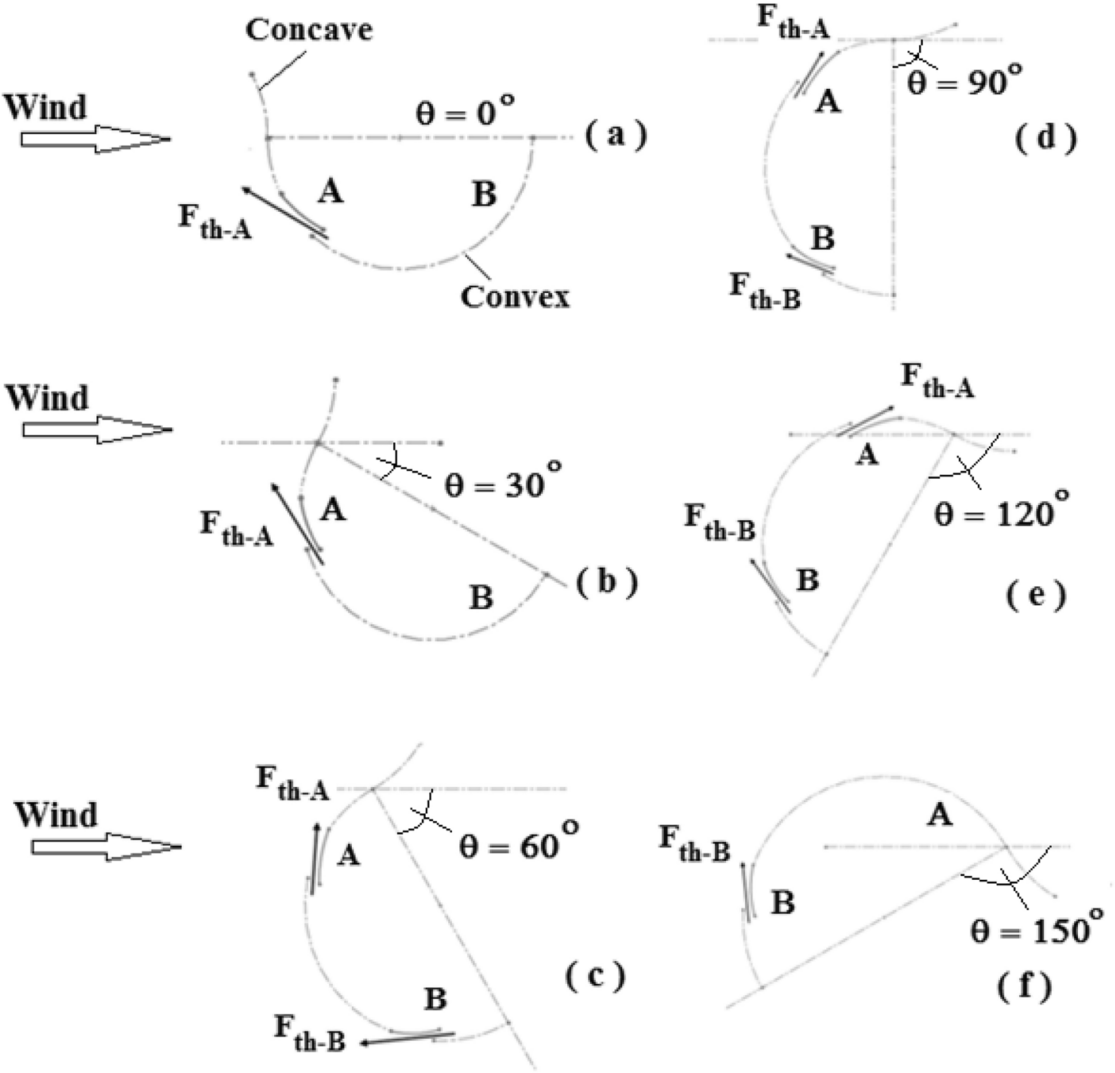
Thrust force produced based on the turbine rotor’s position and the flapping gate’s position on the convex blade surface, with the rotor position ranging from 0 to 150 degrees.

As illustrated in Fig. 3, opening the flapping gate changes the area through which air passes, increasing air velocity at the exit region and producing a thrust force $F_{th}$. This, in turn, creates positive torque acting on the turbine’s axis of rotation. A and B are two flapped gates depicted in Fig. 4. The position of flapped gate A is close to the turbine’s axis of rotation, whereas flapped gate B is close to the blade’s free end. The chance of the two flapping gates A and B opening is controlled by the pressure force P and the centrifugal force $F_{c}$. The openings at each flapped gate control the generated thrust forces $F_{th - A}$ and $F_{th - B}$.If there is a thrust force, it is possible to produce positive torque at the turbine’s axis of rotation.

Due to the convex blade’s angle, there are situations where the convex blade’s torque operating on the turbine axis changes to a positive value instead of a negative one. The convex blade’s negative torque is replaced by a quantity of positive torque added to the concave blade’s torque under these circumstances, which results in a significant improvement in the turbine’s performance and efficiency, both the concave and convex blades cause a positive torque at the turbine axis.

Compared to other modified Savonius turbines described in prior researches, the proposed flapped-gates Savonius turbine design has a number of advantages. These advantages include a simple design, ease of fabrication and installation in confined spaces, the ability to operate under complex wind conditions, independence from wind direction, and good starting performance. An investigation into the potential benefits of using flapping gates Savonius turbine blades is carried out through the present experimental work.

### Experimental study

**Fig. 5:**
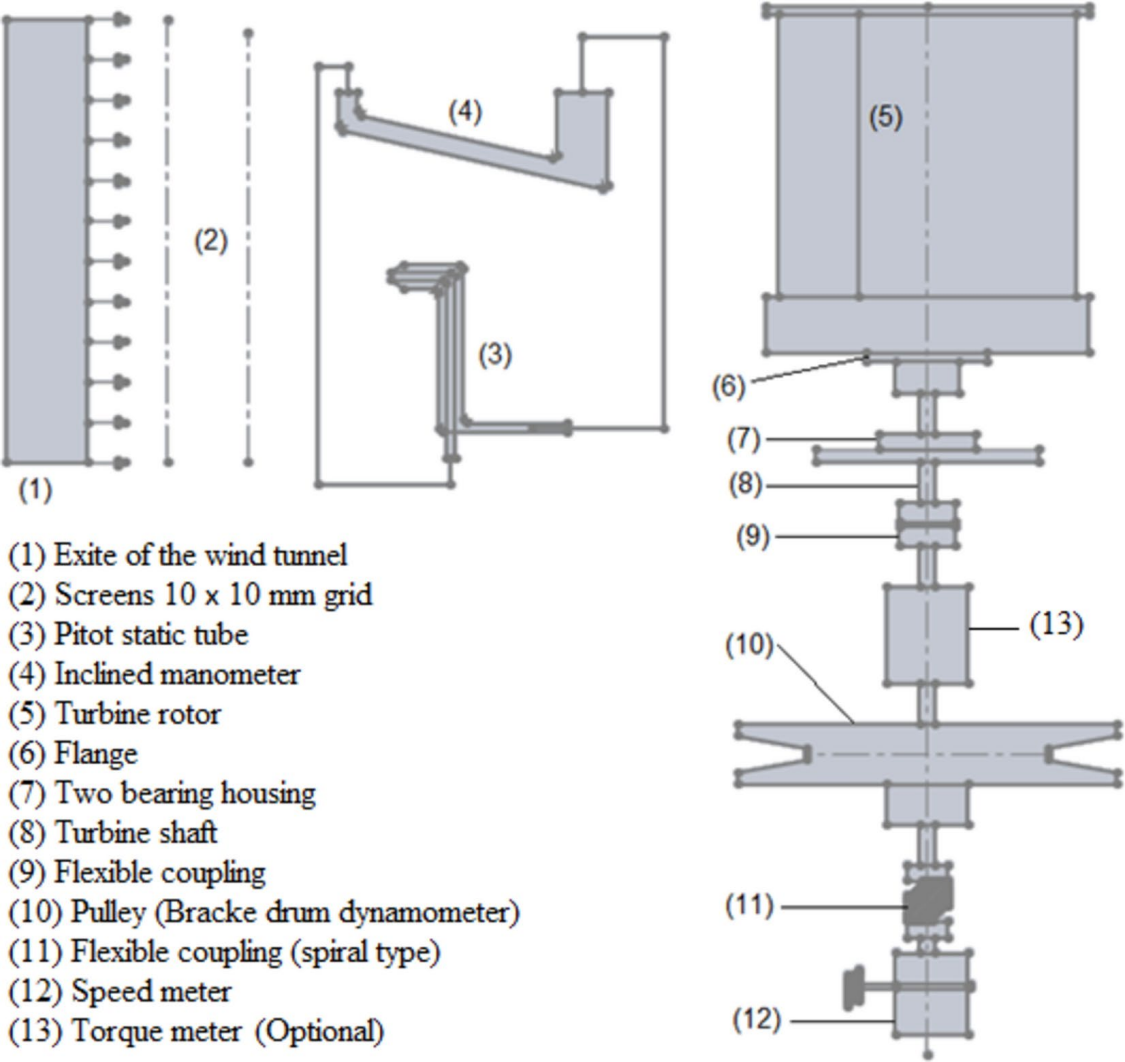
A schematic representation of the key elements that were employed in the second round of experiments to measure the torque, static torque, power, torque coefficient, static torque coefficient, and power coefficient.

An investigation into the potential benefits of using flapping gates Savonius turbine blades is carried out through an experimental study. Based on our current capabilities, it is challenging to construct a modified Savonius turbine blade with a large number of small-area flapping gates originally spread throughout the whole surface of the blade. Thus, two sets of experiments are to be carried out. The first set will determine whether the flapping gate enhances the performance of the traditional Savonius turbine, and if so, by how much. This series of experiments are to be carried out using a small turbine that can be installed within the test section’s open circuit wind tunnel and has one flapping gate in each concave and convex blade. Experiments are to be conducted at various air speed values inside the test section of the wind tunnel. The second set of experiments is to be conducted on a relatively larger Savonis turbine placed behind the exit section of the wind tunnel, after placing a set of screens to improve the air flow condition, which is to lower its turbulence level before entering the Savonius turbine. This is in addition to designing and creating the device on which the Savonius turbine will be installed. This is whether it is normal or modified, with the possibility of placing the measuring devices required to conduct the experiments. This is in addition to those required to carry out the process of increasing the load on the turbine shaft during the operation process. A schematic representation of the experimental setup used in the second set of tests is shown in Fig. 5.

#### Equipments

The main equipments to be used are the low speed wind tunnel of open circuit type, a pitot static probe, a load cell of beam type to measure the tensile force in the highest side of the nylon string, a torque sensor, a load cell of S type to measure the tensile force in the slack side of the nylon string, a structural test bench carrying the Savonius wind rotor and a CPU unit.

#### Wind tunnel

The Hampden Model H-6910–12-150-CDL wind tunnel is equipped with the basic facilities for generating air flows which are to be used in the experiments. The wind tunnel including an inlet cone, a test section of $0.3 \times 0.3\,m$, an outlet cone diffuser with a fan and a main AC circuit breaker. The wind tunnel’s exit section has a circular cross section of 0.7 m in diameter. The air velocity downstream of tunnel exit section could also be changed by the use of suitable turbulence damping screens.

### Pitot static tube (Air velocity measurements)

**Fig. 6:**
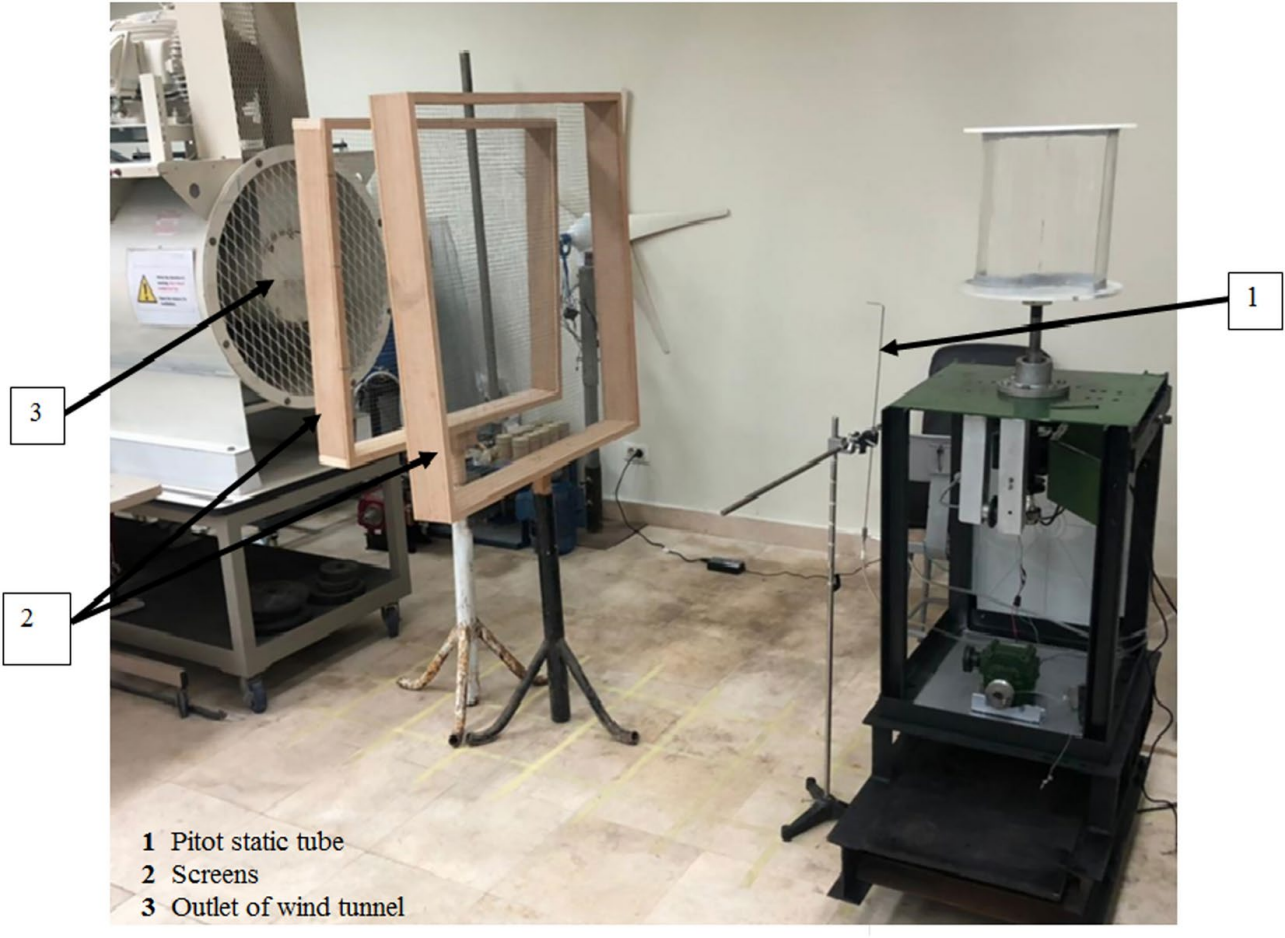
Position of the Savonius wind rotor and the screens downstream from the wind tunnel outflow.

Pitot static tube of a modified Prandtl type is used to measure the total and static pressure at the same point in moving air stream, inside the test section or at the downstream exit section. If the temperature of the fluid is known, the density and flow velocity can be calculated. The air velocity is measured using the pitot static tube connected to an inclined manometer and is controlled by the variation of the speed of the blower fan. The experiments were carried out for different values of the fan speed and the corresponding values of the air velocity were reported. In the second set of experiments, to ensure uniform air flow, the Savonuis wind rotor should be located around 5 m away from the wind tunnel exit. Due to space constraints downstream of the wind tunnel exit in the laboratory, two screens were utilized to ensure uniform air flow, and the wind rotor was installed at a distance of 1 m measured from the last turbulence screen. Screens with a grid of 10 × 10 mm and a space of 120 mm between them are employed as shown in Fig. 6. The Savonuis rotor shaft is supported by two anti-friction bearings in a cantilever arrangement. To reduce the friction torque acting on the rotor, the bearing seals are removed and each bearing is sprayed with W-D 40 (a commercially available spray) lubricant before collecting each reading.

### Load cells and speed sensor (Torque and power measurements)

**Fig. 7:**
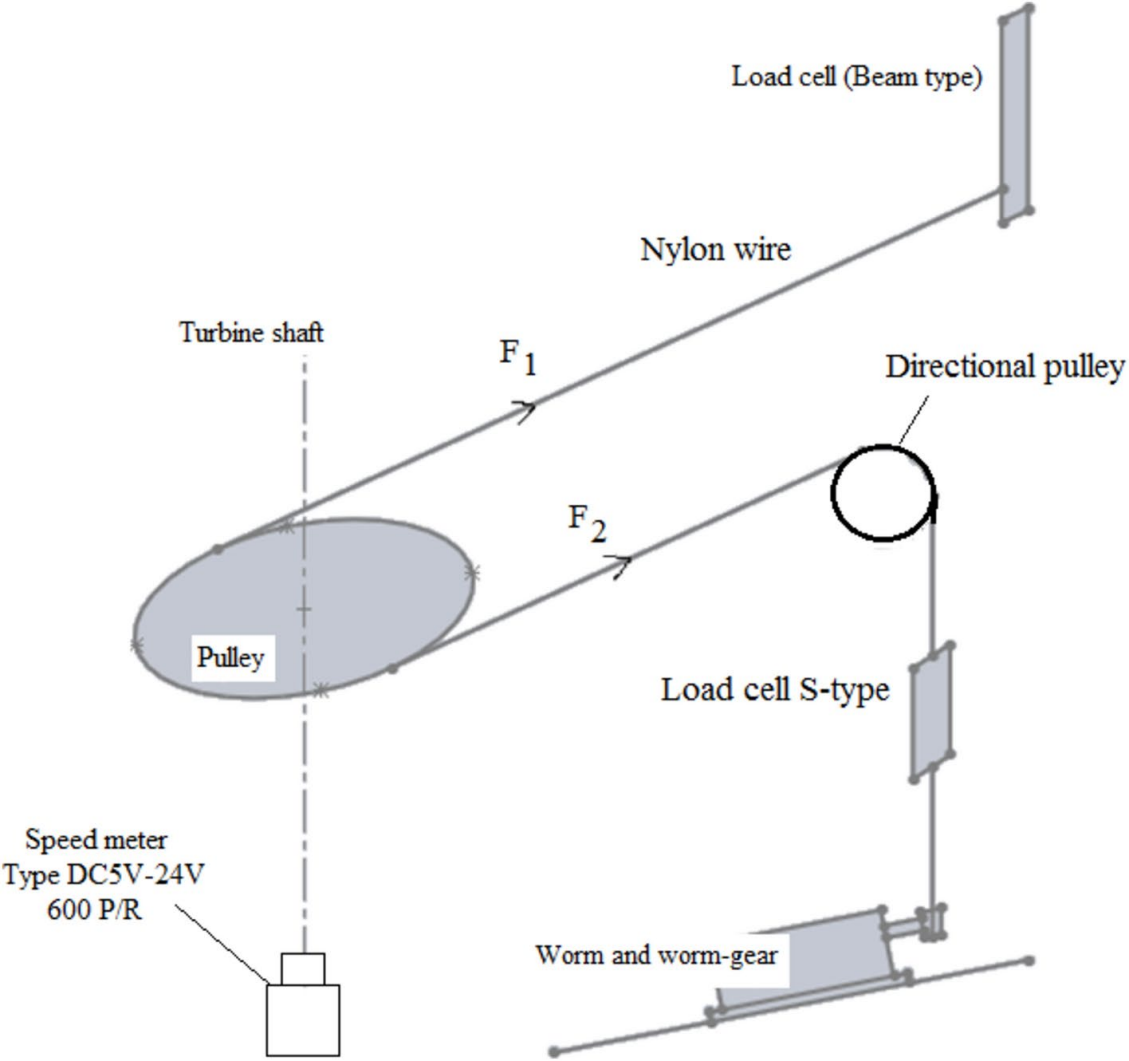
The loading mechanism and the load cells used to measure the force acting on both the tight side F_1_ and the slack side F_2_.

A brake drum (pulley) dynamometer is used to measure the torque of the turbine shaft by creating frictional resistance to the shaft’s motion using a nylon wire. Two load cells were used to measure the forces $F_{1} \,\,and\,\,F_{2}$ in the nylon wire’s tight and slack sides respectively, see Fig. 7. The first load cell is of the beam type with a capacity of 200 N to measure $F_{1}$ acting on the tight side. The second load cell is of S-type to measure the force $F_{2}$ acting on the slack side of the nylon string. The gauges are linked to a Wheatstone bridge as well as a.

HX711 Load Cell Amplifier Interface with Arduino. HX711 was used to magnify the load cell’s output signal. The HX711 is a 24-bit precision analogue to digital converter (ADC) designed for industrial control applications that interface directly with a bridge sensor. Rotational speed of the rotor is recorded by a speed sensor of type DC5V-24V 600P/R.

### Blade description

Conventional Savonius turbine: For the first series of experiments, the traditional Savonius rotor is made up of two semi-cylinder-shaped blades without end plates; for the second set of examinations, it has endplates. The two blades of the traditional Savonius turbine used in the first set of experiments are each half-cylinders of 100 mm in diameter and 100 mm in height. The blades are made from aluminum sheets that are 1 mm thick. In the second set of experiments, the traditional Savonius rotor is made up of two half-cylinder blades, Fig. 10a, that are supported by two endplates, as illustrated in Fig. 11a. The blade’s height after being inserted into the endplates is 324 mm, which is also the diameter of the traditional turbine rotor. The rotor’s S shape was formed using a transparent acrylic sheet that was 4 mm thick, and each endplate has a diameter of 350 mm and a thickness of 4 mm.

**Fig. 8:**
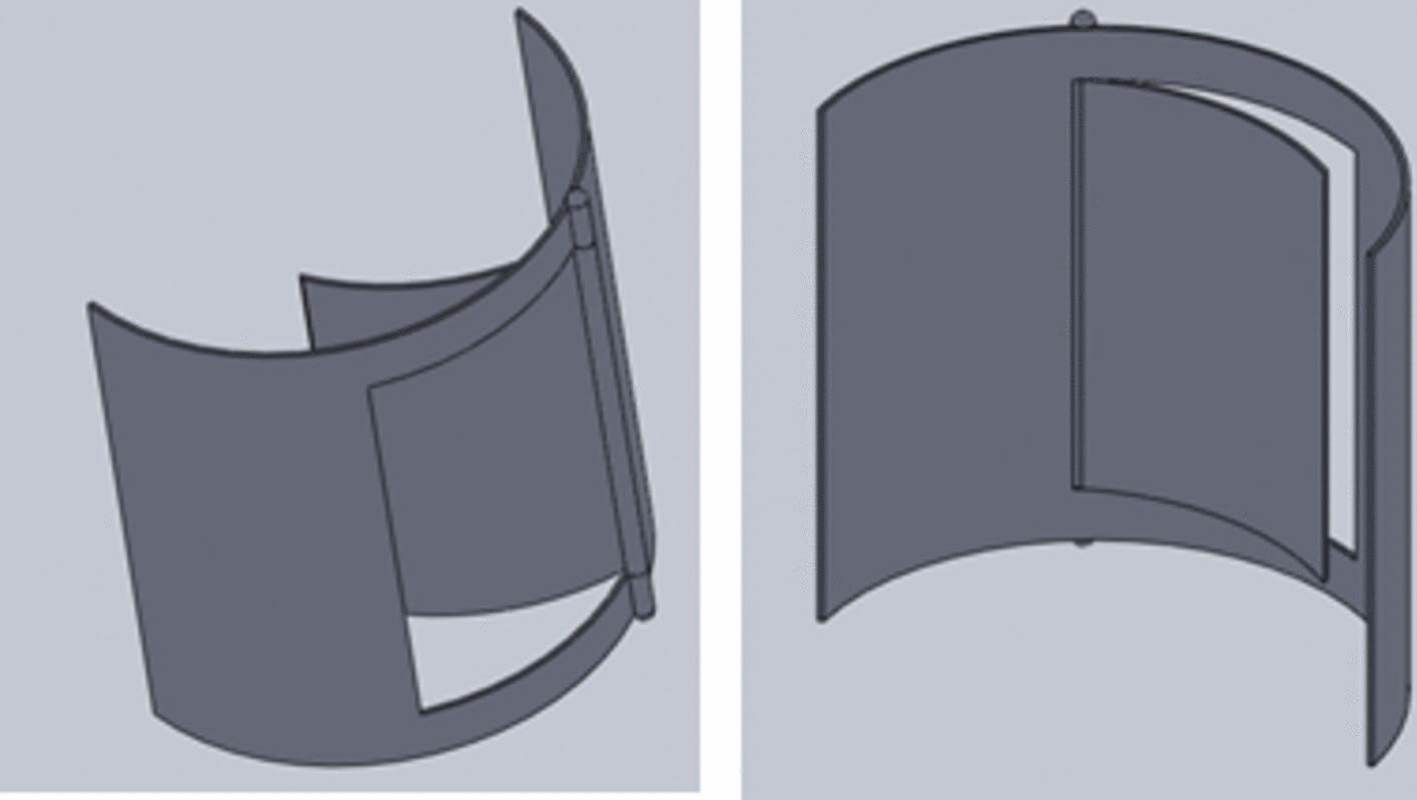
The flapped-gate on each of the Savonius blades.

**Fig. 9:**
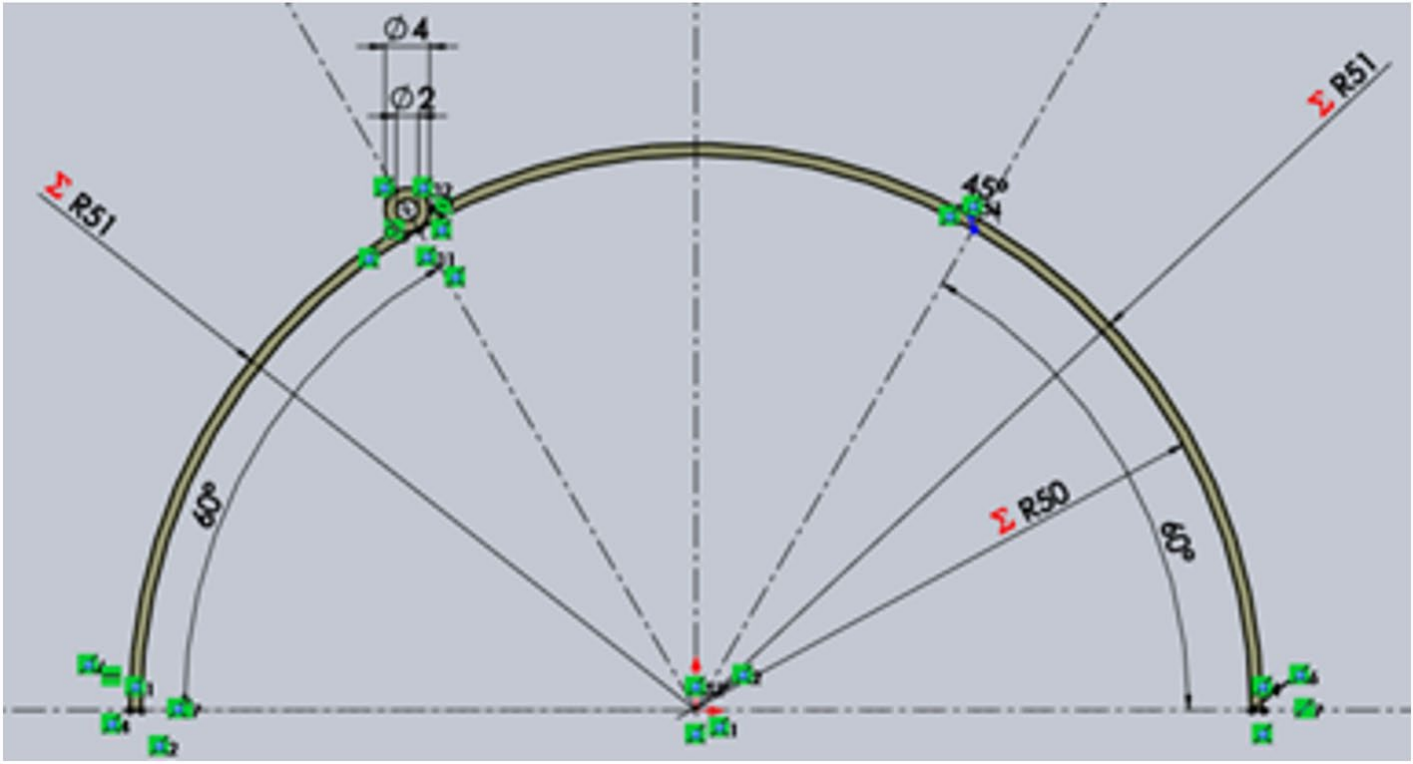
The size of the turbine blade, including the flapped-gate.

**Fig. 10:**
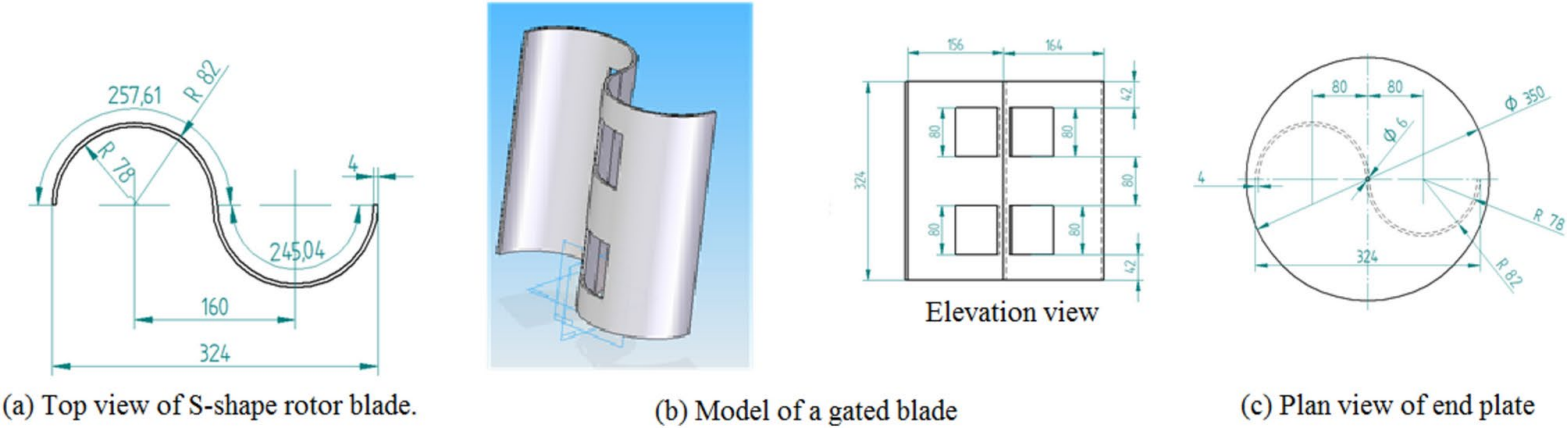
Traditional and modified Savonius blades.

**Fig. 11:**
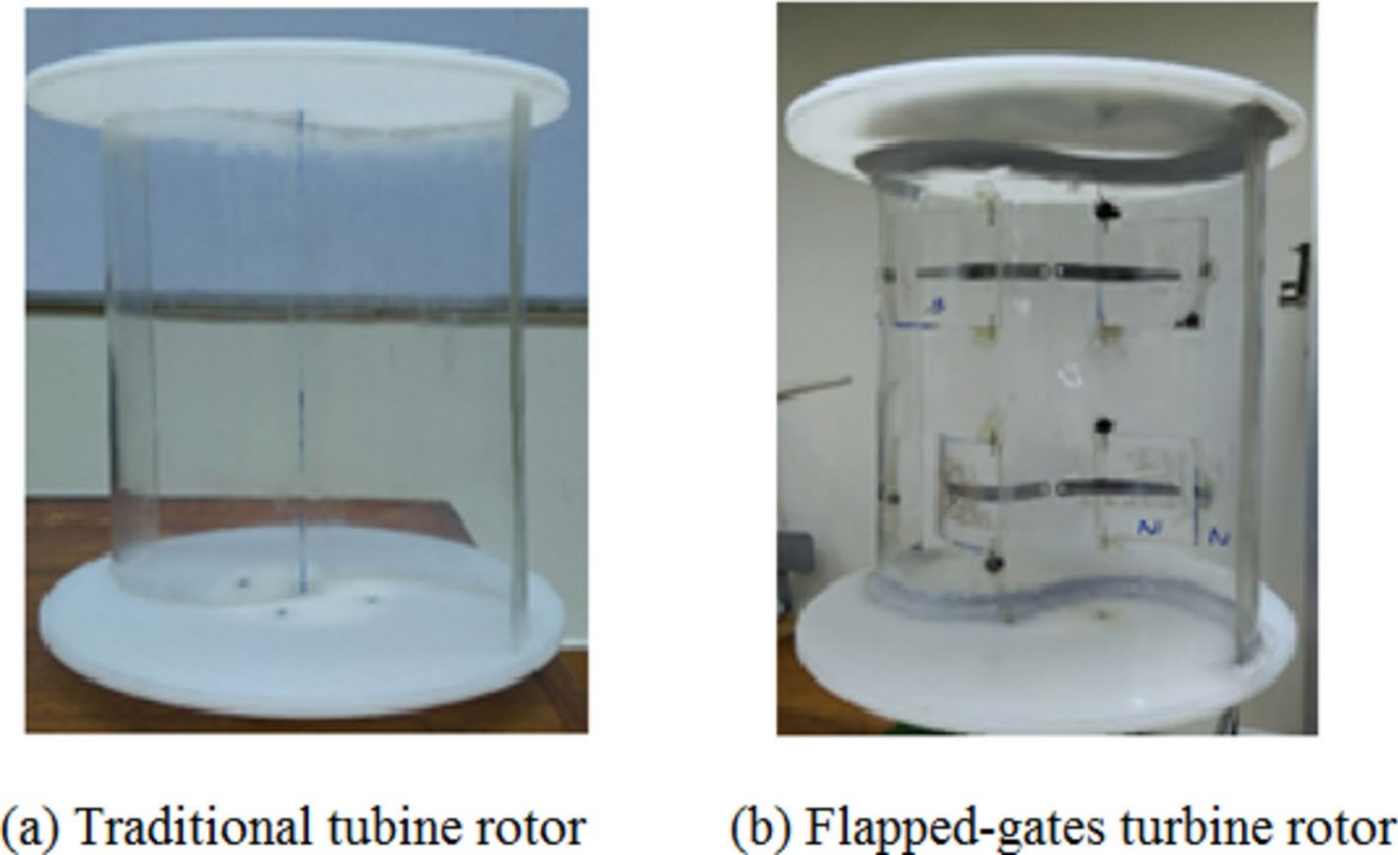
Photographs of the produced traditional turbine rotor (**a**) and the completed flapped-gates turbine rotor (**b**) after manufacturing and assembly.

Modified Savonius turbine: Each Savonius blade has the same dimensions as the conventional one, but as shown in Fig. 8, which shows two perspectives of a single flapped-gate on the turbine blade’s surface, one flapped-gate is used for the first set of experiments within the test section of the wind tunnel; the blade has no endplates, and the flapper rotates around a vertical axis parallel to the turbine’s axis of rotation, with the flapper axis supported near the turbine axis. Figure 9 shows the turbine blade’s size that includes the flapped-gate. The turbine in the second set of experiments has the same primary features as the conventional Savonius rotor in terms of diameter, height, and endplates. However, as illustrated in Fig. 10b, each blade has two flapped-gates, each measure 80 mm in length and 40 mm in arc. To create the flapped-gates, which have two on the concave side and two on the convex side, the flappers—which are composed of the same material as the blade—are secured in place by tiny hinges. The complete flapping gates Savonius rotor is shown in Fig. 11b.

### First set of experiments: verification of the proposed idea

**Fig. 12:**
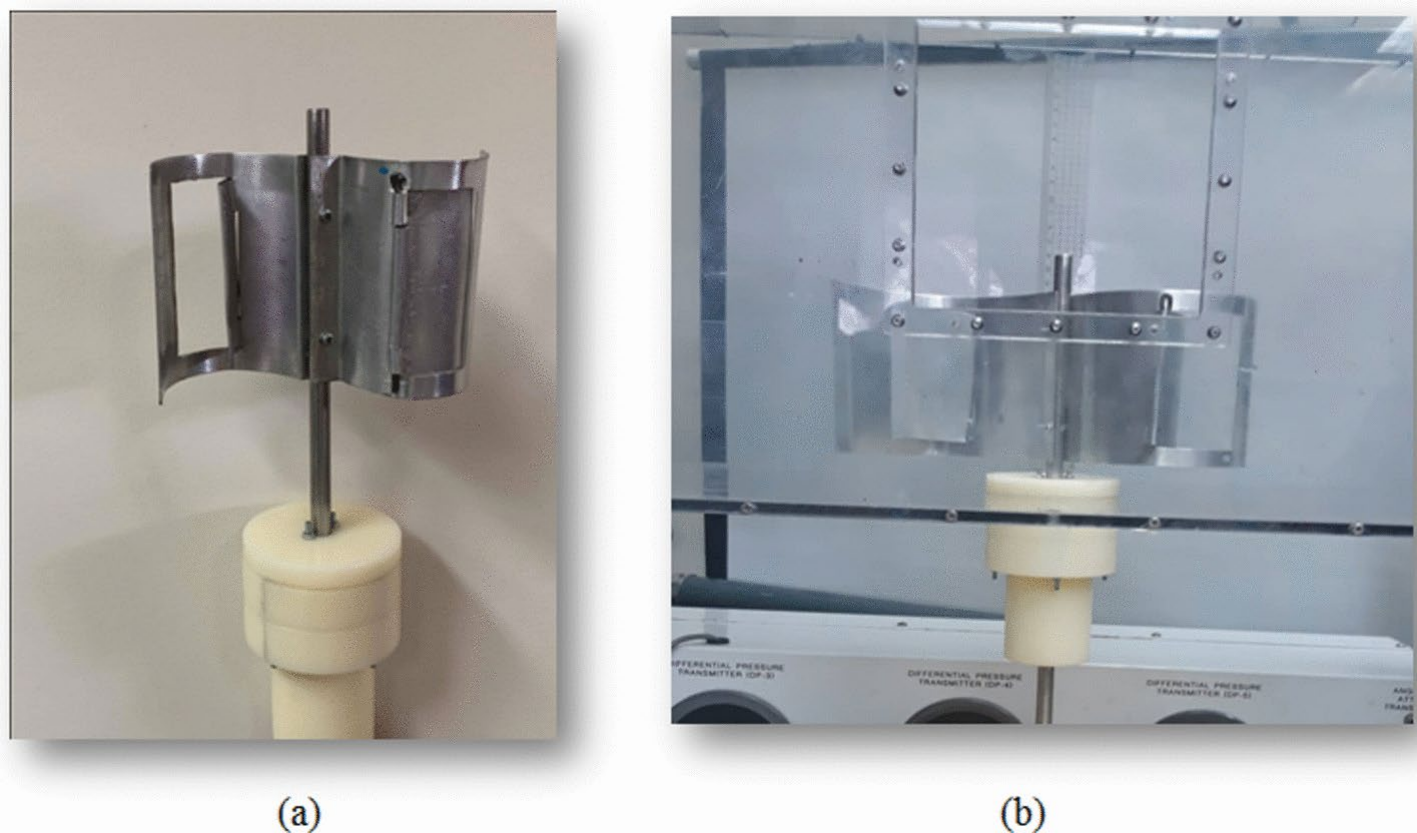
(**a**) The turbine rotor with one flapped-gate in each blade, as well as the turbine shaft support in the two-bearing housing; (**b**) Turbine rotor support within the wind tunnel’s test section.

The conventional Savonius turbine has two blades, each of which is a half-cylinder with a diameter of 100 mm and a height of 100 mm. Aluminum sheets with a thickness of t = 1 mm are used to make the blades. The novel blade configuration is of the same size as the traditional one, but it has a flapped-gate, as shown in Fig. 12. The flapped-gate has an arc length of 52.36 mm and a height of 80 mm, and is positioned in the middle of the turbine’s blade, as shown in Fig. 9. The gate of the concave blade is closed, whereas the gate of the convex blade can be opened to allow air to pass through, resulting in an area change through the gate. Using the structure support as shown in Fig. 16A, the turbine was fixed inside the test section of the wind tunnel. The generator and the turbine shafts are connected, and the generator output is connected to a multimeter so that the voltage (V) and current (I) can be measured. The power $P_{t} = V\,I$ in Watt can be used to calculate the power generated. For different values of air velocity, the power is estimated for both the traditional and flapped-gates Savonius turbines.

### Second set of experiments: turbine performance using the proposed model

**Fig. 13:**
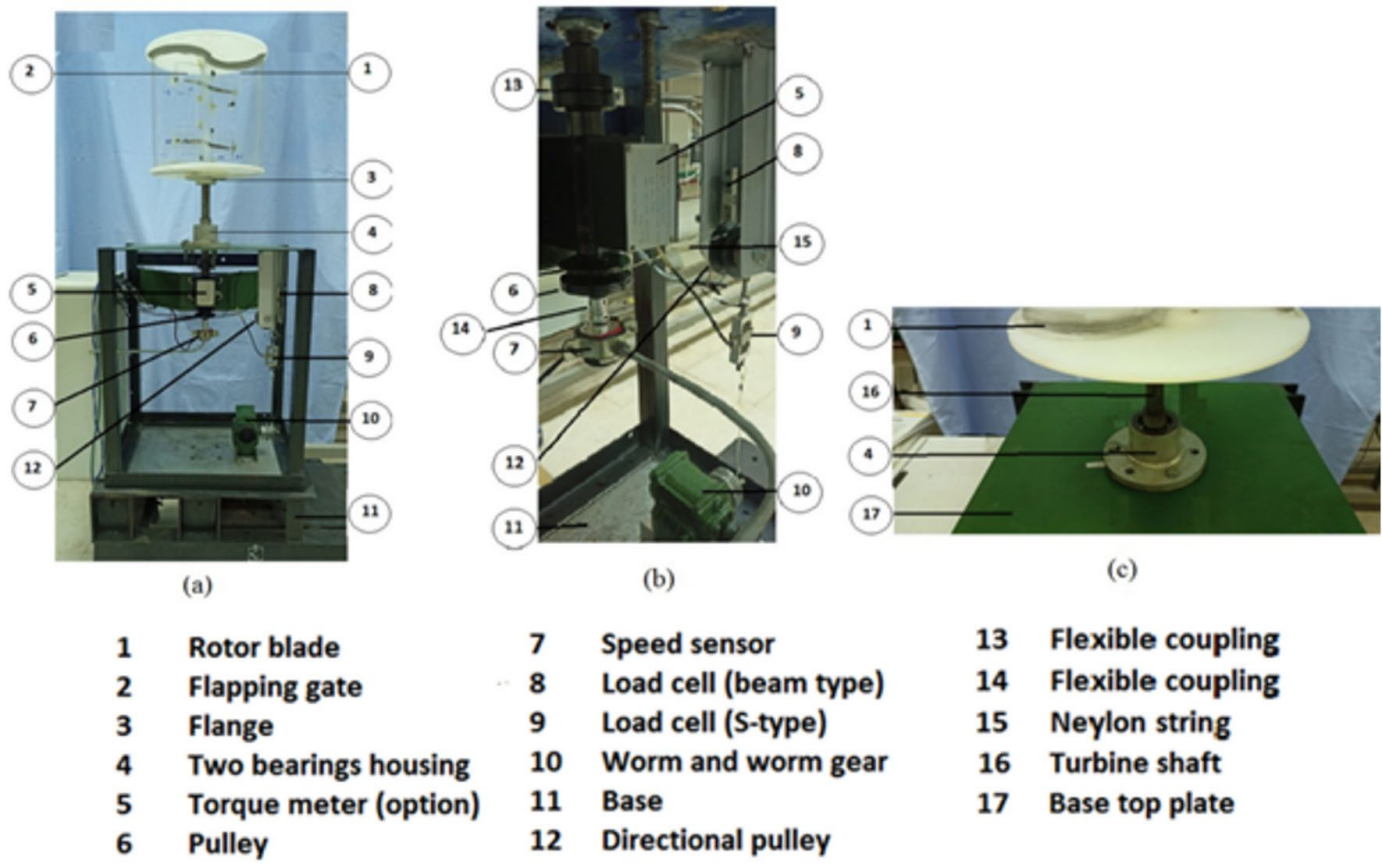
A photograph of the test bench used in the second set of experiments.

**Fig. 14:**
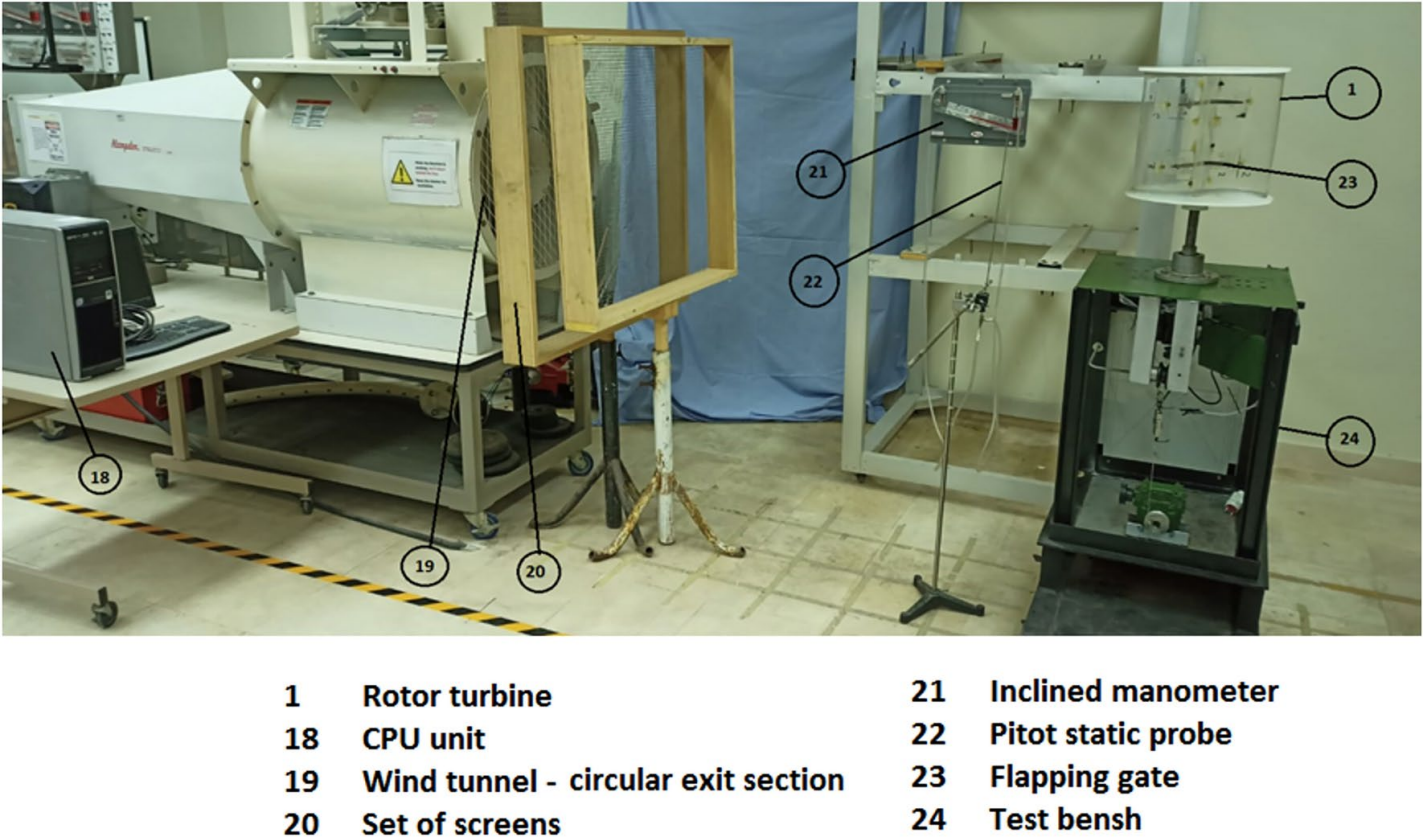
The layout of the experiment components for the second set of experiments.

In the second set of experiments, the static torque generated by Savonius blade rotors for various angular positions and wind speeds is measured. Both regular and modified Savonius blade rotors are used in the studies. Figure 13 depicts the test bench that was designed, manufactured, and assembled. Figure 13a shows a photograph of the test bench used in the second set of experiments, identifying the key elements used to load the turbine shaft as well as the sensors used to detect the various parameters throughout the experimentations. Figure 13b depicts the loading mechanism and the speed sensor. The photo in Fig. 13c shows the two-bearing housing holding the turbine shaft after removal of the bearing seals, as well as the upper plate of the construction and how the rotor blade is supported. Figure 14 shows a photograph of all of the elements and the layout of the experiment components for the second set of experiments.

#### Experiment (2–1): bearing friction and aerodynamic effect

The first group of tests is carried out to determine one of the important characteristics of the test bench at different operating wind speeds. The friction in bearings, aerodynamic effects, friction power loss and free braking time are to be evaluated in order to understand the behavior of the system at different values of wind speed. The test procedure for experiment (2–1) may be summarized as follows:

- 1. Disconnecting the resisting mechanism at the end of the rotor assembly
- 2. Starting the wind tunnel and controlling the speed of the fan.
- 3. Adjusting the speed of fan and measure the wind speed.
- 4. Using the speed sensor attached to the rotor to measure the rotor speed at no load till reaching the steady state condition, $\omega_{\,1}$ at time $t = 0$
- 5. Stopping the stream of the turbine rotor by cutting the air flow using a movable barrier.
- 6. Recording the variation of the rotor speed with time and estimate the free braking time $t_{o}$ at $\omega_{2} = 0$.
- 7. Determining the average value of angular acceleration of the rotor $\alpha_{f} = \frac{{\omega_{\,1} - \omega_{\,2} }}{{t_{o} }} = \frac{{\omega_{\,1} }}{{t_{o} }}$.
- 8. Estimating the friction and aerodynamic resistance torque $T_{f} = - \alpha_{f} \,J_{eq}$; where $J_{eq}$ is the polar moment of inertia of the rotor in $kg\,m^{2}$.
- 9. Repeating the steps 4–6 at different operating wind speeds and reporting the outputs.

#### Experiment (2–2): static torque produced by savonius rotor for different angular position at various wind speeds.

A set of experiments were carried out to evaluate the static torque produced by traditional and modified wind mill. Using the brake drum measuring system, Fig. 7, a set of tests were performed to evaluate the static torque and static torque coefficient of the rotor at a given rotor angle. The static torque of the rotor is measured at every 30° of the rotor angle, and the use of a worm gear box made the loading of the rotor easy to obtain and control at any given wind velocity. The test procedure for experiment (2–2) may be summarized as follows:

1. Adjusting the rotor blades to be at the specified position 0,30,60,90,120,150 and 180 deg.
2. Rotating the worm to increase the forces $F_{1} \,\,and\,\,F_{2}$ loading the break drum sufficiently to prevent rotation at a specified value of air velocity and a certain rotor position.
3. Starting the wind tunnel and adjusting the speed of the fan to obtain the required value of the air velocity downstream from the wind tunnel exit section.
4. Using the pitot static tube which is connected to the inclined manometer to measure the velocity of the free air stream upstream of the rotor position.
5. Making a fine turning for the fan speed to get the desired value of the free air velocity.
6. Gradually releasing the load until the rotor begins to move. At that instance, $F_{1}$ and $F_{2}$ forces are to be measured and recorded using the load cells.
7. Determining the values of static torque $T_{s}$ and static torque coefficient $C_{ts}$ using the relations: $T_{s} = (F_{1} - F_{2} )\,\left( {\frac{{D_{pully} + D_{wire} }}{2}} \right)$ and $C_{ts} = \frac{{4\,T_{s} }}{{\rho \,U^{2} D^{2} H}}$.
8. Repeating the experiment for different angular position at various wind speeds

#### Experiment (2–3): torque coefficient and power coefficient

A series of experiments are conducted to determine the torque, torque coefficient, power, and power coefficient produced by conventional and modified rotors at various wind speeds. The procedure of the experiment may be summarized as follows:

1. To set the rotor at the desired position
2. To set the pitot static probe upstream of the rotor to measure the velocity of the stream of the air
3. To start the wind tunnel and adjust the speed of the fan to obtain the required air velocity.
4. To adjust speed of the rotor by changing the resisting moment acting on the rotor by changing the forces $F_{1}$ and $F_{2}$ through the rotation of the worm
5. To measure the values of rotor speed in RPM, the force $F_{1}$ and $F_{2}$ in tight side and slack side of the nylon string, respectively.
6. To calculate the value of the torque T, $T = (F_{1} - F_{2} )\,\left( {\frac{{D_{pully} + D_{wire} }}{2}} \right)$.
7. To calculate the tip speed ratio, $TSR = \frac{\omega \,(D/2)}{U}$, where $\omega = \frac{2\,\pi \,N}{{60}}\,\,.$
8. To calculate the torque coefficient and power coefficient using the relations:
  $$C_{t} = \frac{4\,T}{{\rho \,U^{2} D^{2} H}}\,\,\,\,and\,\,\,\,C_{p} = TSR\, \times \,C_{t} \,\,.$$
9. To repeat the steps from 4 to 9 for various values of wind speed ranging from 5 m/s to 9 m/s. T
10. The findings can be obtained through adjusting the braking drum system using the worm-gearbox to change rotor speed. The torque coefficient and power coefficient are to be determined for rotor tip speed ratio ranging from 0.2 to 1.2 and air velocity ranging from 5 to 9 m/s.

### Data reduction

- The turbine’s performance can be expressed in terms of dimensionless quantities based on the outcomes of the experiments. The data reduction can be introduced by the following dimensionless quantities:
- Reynolds number is calculated based on the diameter of the rotor D using the following definition $R_{e} = \frac{\rho \,U\,D}{\mu }$ with $\rho = 1.225\,{{\,\,\,kg} \mathord{\left/ {\vphantom {{\,\,\,kg} {m^{3} }}} \right. \kern-0pt} {m^{3} }}$. U is the free stream air velocity in m/s, D is the rotor diameter of 0.324 m and $\mu$ is the absolute viscosity of air, $\mu = 1.525 \times 10^{ - \,5} \,Pa\,s\,\,at\,\,20\,\,{}^{o}C$.
- Tip speed ratio is given by $TSR = \frac{\omega \,D}{{2\,U}}$; where $\omega$ is the angular velocity of the rotor.
- To determine the torque coefficient, use $C_{t} = \frac{4\,T}{{\rho \,U^{2} D^{2} H}}$; where H is the turbine height of 0.324 m and the torque $T_{r}$ is the resisting load acting on the rotor of the turbine calculated as $T_{r} = (F_{1} - F_{2} )\,\left( {\frac{{D_{pully} + D_{wire} }}{2}} \right)$. The diameter of the pully is 0.05 m and the diameter of the wire is 2 mm.
- Power coefficient can be obtained using $C_{p} = TSR\, \times \,C_{t}$.
- Balance ratio B is given by $B = \frac{D\,\,H}{{A_{exit} }}$. $A_{exit}$ is the area of the wind tunnel exit section. The effect of blockage ratio is negligible on $C_{p} ,\,C_{t} \,\,and\,\,C_{ts}$ for open wind tunnel as reported by Kemoji [47]. In the present study, blockage ratio is negligible where the tests are out in downstream of the wind tunnel exit section.
- Aspect ratio is the ratio of the rotor height to rotor diameter, $AR = \frac{H}{D}\,\,.$

## Results and discussion

The first set of experiments were conducted to validate the concept of the suggested modification to the Savonius turbine blade, which contains a single flapping gate that revolves about an axis parallel to the turbine’s axis of rotation. The turbine is installed in the wind tunnel’s test section area, and its output power is to be estimated. The data is gathered and reported in Table 4, where the percentage power difference is indicated as follows,

$$\% P_{t} = \frac{{P_{t - \bmod ified} - P_{t - traditional} }}{{P_{t - traditional} }}\,\, \times \,\,100$$

**Table 4:** The findings from the initial series of tests conducted in the test portion of the wind tunnel.

Air velocity (m/s)	Generated Power ($\mu \,W$)		$\% P_{t}$
	Traditional	Flapped-gates	
6.3	6.96	8.19	17.67241
8.4	15.24	17.2215	13.02493
9.5	19.782	22.98	16.16621
11.5	28.509	33.66	18.06798
12.6	37.316	37.316	0
14.2	49.456	49.456	0

The findings reveal that using a modified Savonius turbine with flapping gates increases output power by an average of 16.233 percent over velocity ranges of 6.3 to 11.5 m/s. The percentage starts at 17.572% at an air velocity of 6.3 m/s; increasing the air velocity value causes an increase in the rate of rotation of the rotor, which impacts the opening of the flapping gates as the centrifugal force increases. When the air velocity is sufficiently high, the centrifugal force exceeds the pressure force, and the gate closes. The modified blade then performs similarly to the traditional blade.

The second set of tests involves changing the blade shape to a half-cylinder with a diameter of 162 mm and a height of 324 mm. S-shape of the original and modified Savonius rotor has a rotor aspect ratio of $AR = {H \mathord{\left/ {\vphantom {H {D = }}} \right. \kern-0pt} {D = }}1$. The initial group of experiments, Experiment (2–1), was carried out to evaluate the friction in bearings, aerodynamic effects, friction power loss, and free braking time to understand the behavior of the system at different wind speeds. The Savonuis rotor shaft is supported in a cantilever configuration by two bearings with extremely low friction. The experiments were carried out for the typical Savonius rotor before removing the seals of the bearings, and after their removal and spraying each bearing with a lubricant before each reading. Table 5 presents the rotor speed and free breaking time for the two examinations. The findings of the experiment demonstrate that it is essential to lower the friction torque acting on the rotor by removing the bearing seals and spraying each bearing with W-D 40 (an available commercial spray) lubricant before each reading.

**Table 5:** Bearing friction and aerodynamic effects using traditional rotor.

$U\,\,(m/s)$	Bearings with the seal			Bearing without the seal		
	N $$(RPM)$$	$$t_{o} \,\,(s)$$	$$(1/s^{2} )$$$$\alpha_{f}$$	N (RPM)	$$t_{o} \,\,(s)$$	$$(1/s^{2} )$$$$\alpha_{f}$$
5.7	55	2.6	2.304	350	69.95	0.523
5.92	75	3.68	2.134	360	73.72	0.516
6.995	161	5.84	2.887	395	81.08	0.51
7.28	216	7.73	2.926	434	88.84	0.512
8.32	294	10.29	2.992	470	89.49	0.549

$\alpha_{f}$ is the angular acceleration of the rotor, $t_{o}$ is the breaking time.

**Fig. 15:**
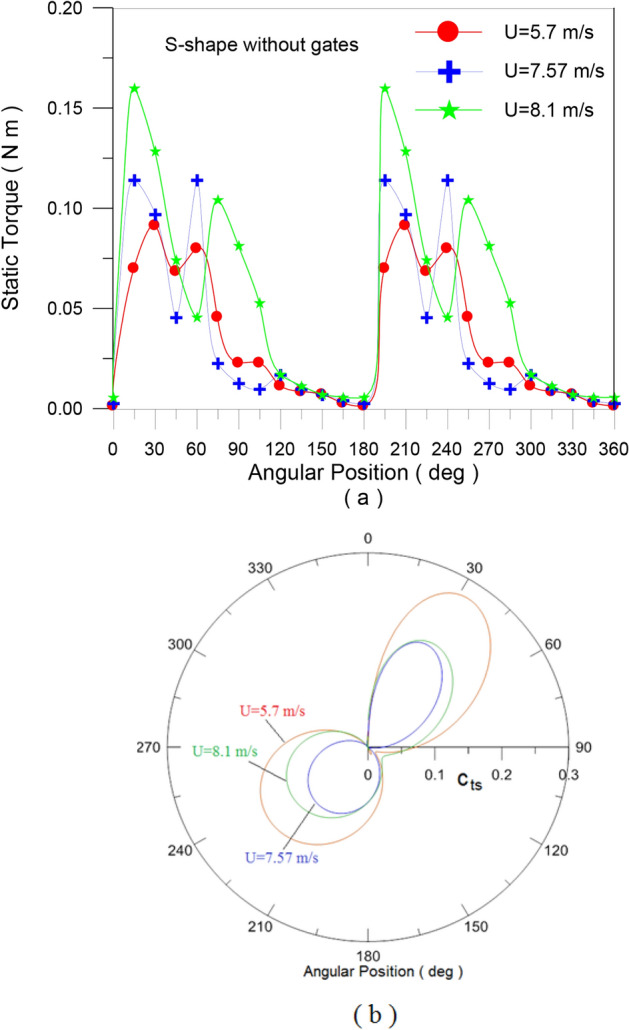
Static torque and static torque coefficient versus the angular position of the traditional S-shape rotor for different values of the wind speed.

**Fig. 16:**
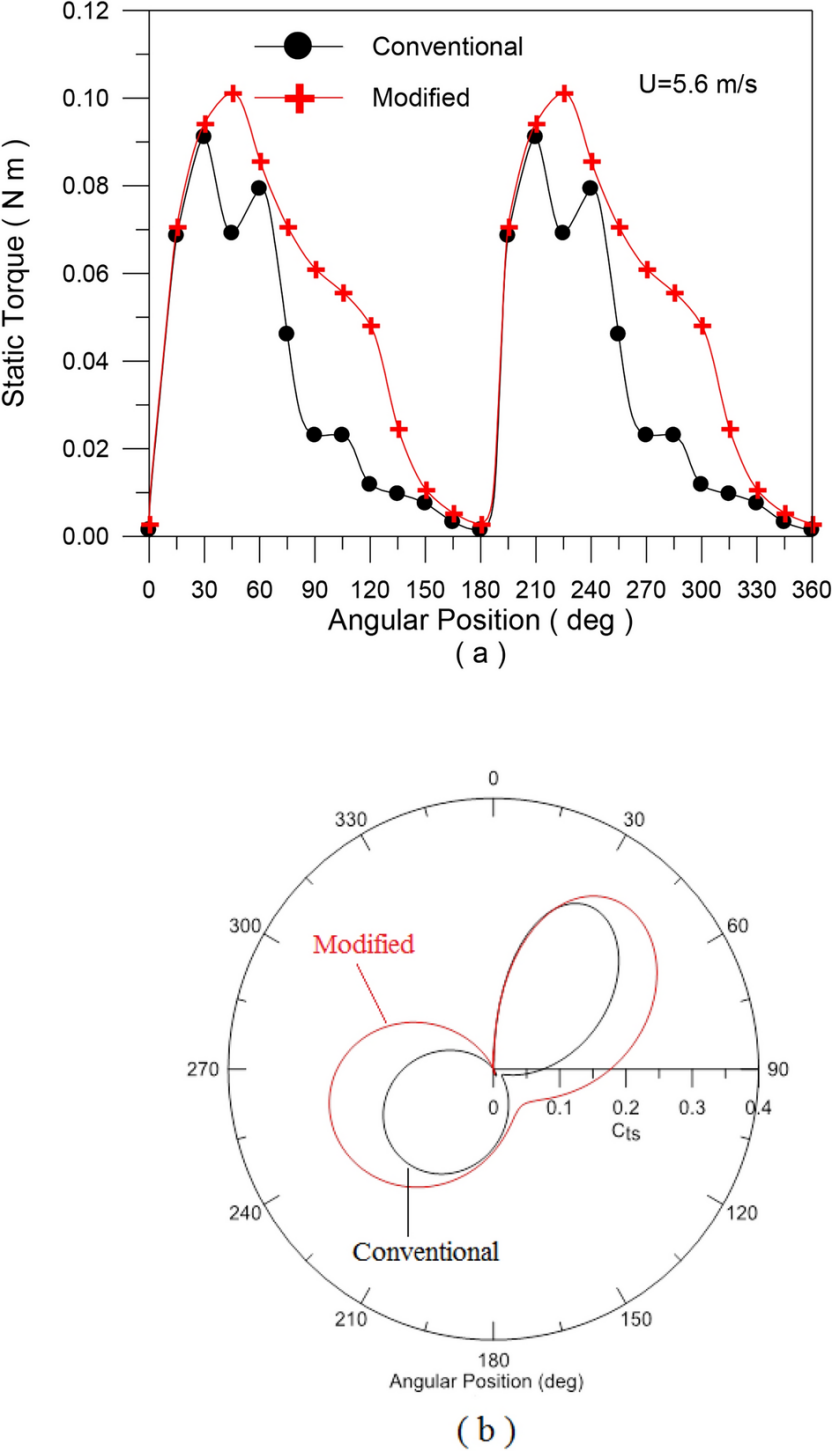
Static torque and static torque coefficient versus the angular position of the traditional and modified turbine rotors at wind speed of U = 5.6 m/s.

**Fig. 17:**
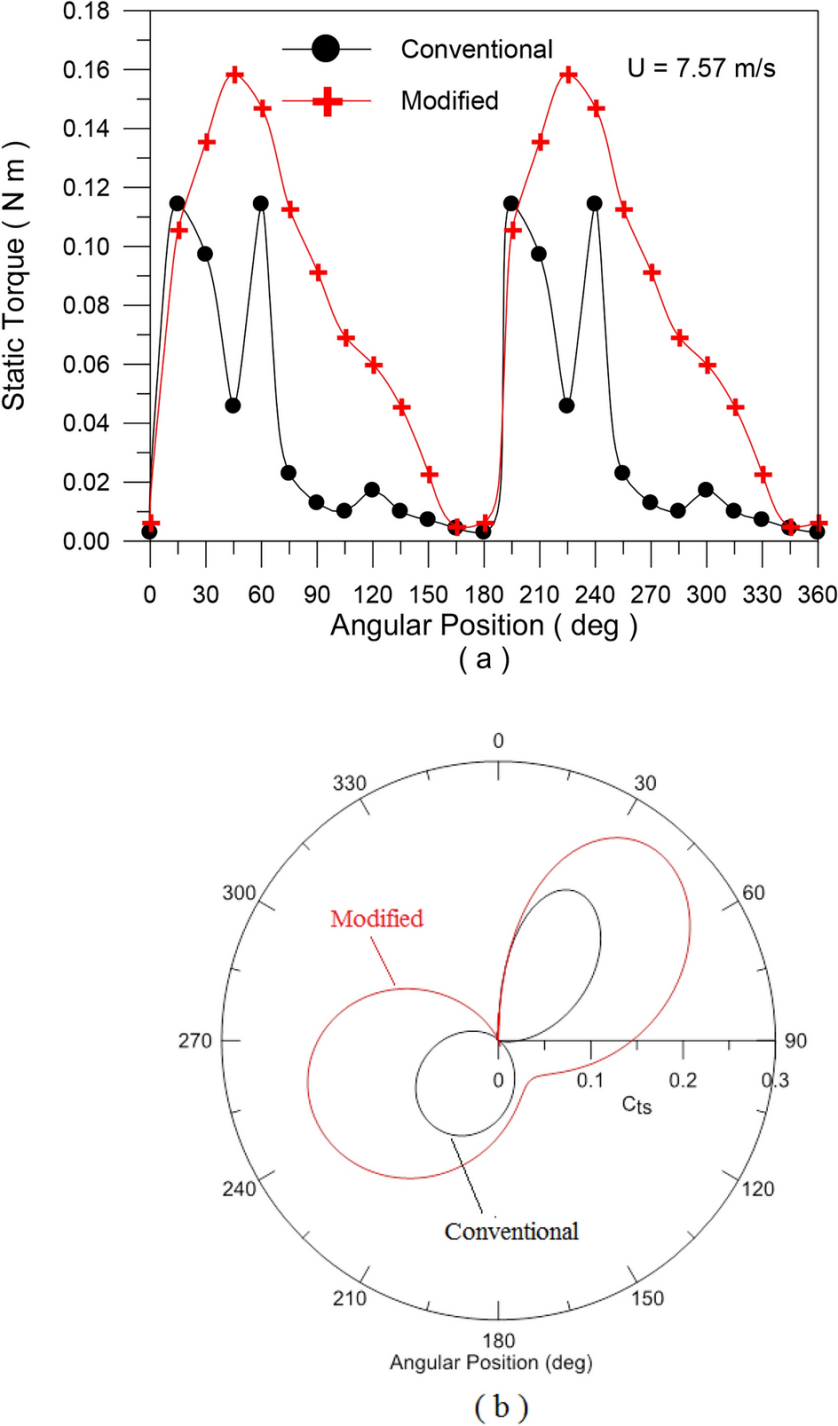
Static torque and static torque coefficient versus the angular position of the traditional and modified turbine rotors at wind speed of U = 7.57 m/s.

In the second set of experiments, Experiment (2–2), a series of tests were carried out on both traditional and modified rotors to determine the static torque and static torque coefficient of the rotor at a specific angle. For a conventional rotor, Fig. 11a, the static torque is measured for every 30 degrees of rotor angle. Figure 15a shows how the static torque fluctuates with different wind speeds (U = 5.7, 7.57, and 8.1 m/s), whereas Fig. 15b shows how the static torque coefficient varies with turbine angular position for the same wind speed. The obtained experimental points are fitted with the 6^th^ polynomial fit. The results are in good qualitative match with previous investigations. Static torque and its coefficient behave in a similar way to angular position, but their values vary depending on wind speed. For both the conventional savonius rotor and the modified flapped-gates Savonius turbine at air speeds of 5.6 m/s and 7.57 m/s, Figs. 16 and 17 depict the variation of the static torque and static torque coefficient at various turbine locations from 0 to 360 degrees at wind speeds U = 5.6 m/s and 7.57 m/s. Compared to the old design, the new one has a higher available static torque and coefficient of static torque. This demonstrates the design’s effectiveness.

**Fig. 18:**
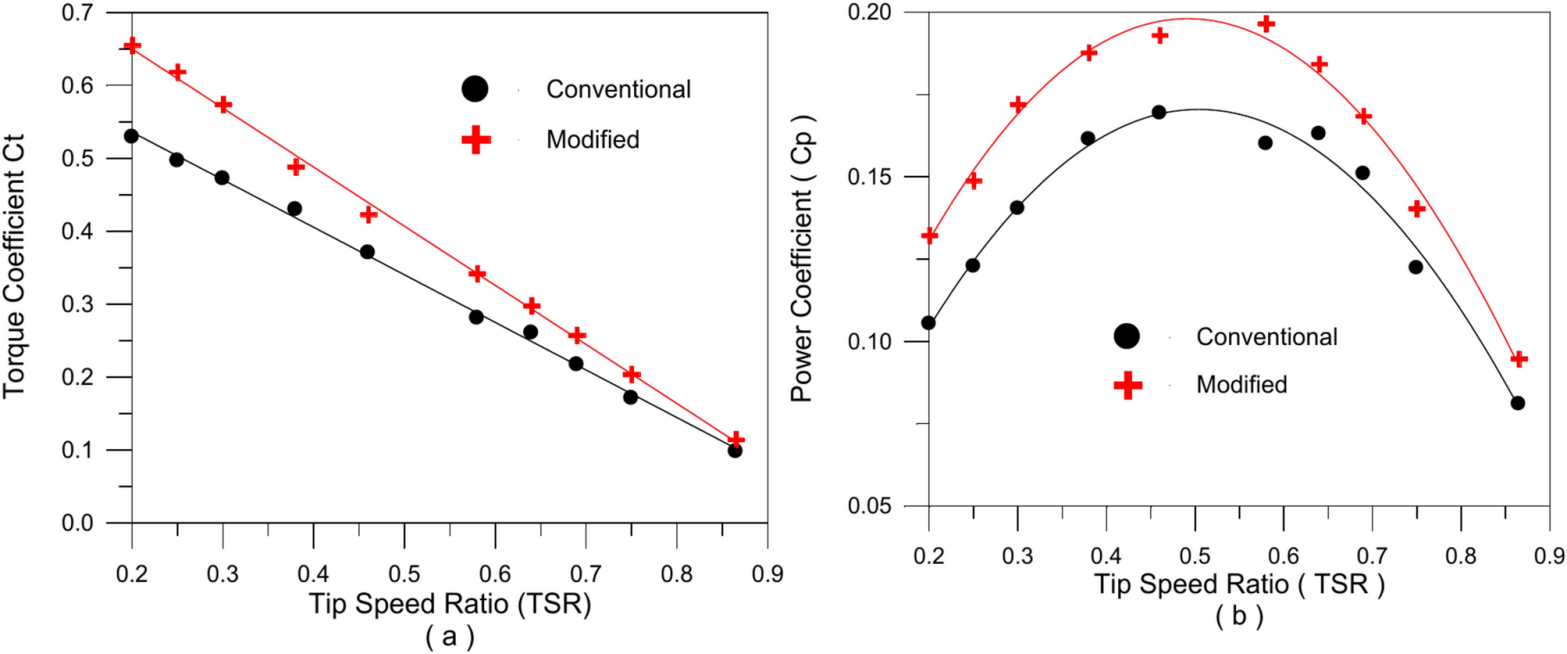
The torque coefficient (**a**) and power coefficient (**b**) variations with tip speed ratio for both traditional and modified turbine rotors.

A set of tests are carried out in Experiment (2–3) in order to evaluate the power, power coefficient, torque, and torque coefficient produced at different wind speeds by both modified and standard rotors. After the torque and power coefficient values for the conventional turbine are compared to numerous earlier studies, a good agreement is found. Compared to the original design, the new one has a higher available static torque and coefficient of static torque, as shown in Figs. 16 and 17. This demonstrates the design’s effectiveness. For both the conventional turbine and the turbine with the modified blade, Fig. 18 illustrates how the torque and power coefficients change with the tip speed ratio. The torque coefficient decreases as the tip speed ratio increases, Fig. 18a, but it is higher with the modified blade geometry than with the conventional one. At a tip speed ratio of 0.2, the torque coefficient improves by 23.833%, and at a tip speed ratio of 0.865, it improves by 16.668%. On the other hand, the power coefficient was improved roughly by 16.914% at higher tip speed ratio of 0.865, according to the data on the change of the power coefficient in Fig. 18b, which shows an average increase of 25% at middle speed ranges. The flapping gates open and air flows through the holes in the convex side of the blade when it is on the windward side, reducing the negative torque operating on the turbine rotor and increasing the overall torque. This was good since the gates opened less widely due to the stronger centrifugal force acting on them.

Static testing, which disregards centrifugal force, demonstrated the design’s effectiveness. To reduce the effects of centrifugal force, it is advised that the mass of the gates should be kept as low as possible.

## Conclusions

The current research offers an innovative design that consists of lightweight flapping gates that open when the convex side is facing the wind source in order to reduce negative torque and improve the efficiency of Savonius wind turbines. Very light leaf or torsional springs can be employed to ensure that the gates close smoothly by returning them to their closed condition. When the flapping gates open, less drag force and resistive torque are transferred to the turbine shaft, increasing the turbine’s efficiency. To do this, two turbines were designed, one with flapping gates and the other with the conventional shape. Then, under the same circumstances, the two turbines were examined. A variety of sensors were used in an experimental setup to gather the required readings. To determine the static torque coefficient and power coefficient, experiments were conducted at different air velocities. It was demonstrated that the new design outperforms the traditional design due to its higher static torque and power coefficient. The following is a summary of the main conclusions:

- The use of the modified flapping gates Savonius turbine blades resulted in a significant increase in the effectiveness of the conventional Savonius turbine. When the blade is on the windward side, the flapping gates open and air passes through the holes in the convex side, decreasing the negative torque acting on the turbine rotor and raising the total torque.
- At higher tip speed ratios, the power coefficient improves only by around 16.914%, but at medium speed ranges, it increases by an average of 25%.
- However, more comprehensive research is required to determine the ideal mass, size, and distribution of the gates all over the entire surface of the Savonius turbine blade.

## Data Availability

All data generated or analyzed during this study are included in this published article, no supplementary information files.
